# Analysis of a Family with Brugada Syndrome and Sudden Cardiac Death Caused by a Novel Mutation of SCN5A

**DOI:** 10.1155/2022/9716045

**Published:** 2022-04-28

**Authors:** Yao-Bin Zhu, Jian-Hui Zhang, Yuan-Yuan Ji, Ya-Nan Hu, Han-Lu Wang, Dan-Dan Ruan, Xiao-Rong Meng, Xin-Fu Lin, Jie-Wei Luo, Wei Chen

**Affiliations:** ^1^Department of Traditional Chinese Medicine, The First Affiliated Hospital, Fujian Medical University, Fuzhou 350001, China; ^2^Shengli Clinical Medical College of Fujian Medical University, Fuzhou 350001, China; ^3^Fujian Provincial Hospital, Fuzhou 350001, China

## Abstract

**Background:**

Brugada syndrome is a hereditary cardiac disease associated with mutations in ion channel genes. The clinical features include ventricular fibrillation, syncope, and sudden cardiac death. A family with Brugada syndrome with sudden cardiac death was analyzed to locate the associated mutation in the *SCN5A* gene.

**Methods and Results:**

Three generations of a Han Chinese family with Brugada syndrome were recruited in the study; their clinical phenotype data were collected and DNA samples extracted from the peripheral blood. Next-generation sequencing was carried out in the proband, and candidate genes and mutations were screened using the full exon capture technique. The family members who participated in the survey were tested for possible mutations using Sanger sequencing. Six family members were diagnosed with Brugada syndrome, including four asymptomatic patients. A newly discovered heterozygous mutation in the proband was located in exon 25 of SCN5A (NM_000335.5) at c.4313dup(p.Trp1439ValfsTer32). Among the surviving family members, only those with a Brugada wave on their electrocardiogram carried the c.4313dup(p.Trp1439ValfsTer32) variant. Bioinformatics prediction revealed that the frameshift of the c.4313dup (p.Trp1439ValfsTer32) mutant led to a coding change of 32 amino acids, followed by a stop codon, resulting in a truncated protein product.

**Conclusion:**

The newly discovered mutation site c.4313dup(p.Trp1439ValfsTer32) in exon 25 of SCN5A may be the molecular genetic basis of the family with Brugada syndrome.

## 1. Introduction

Brugada syndrome (BrS) is a disorder of cardiac electrical activity with genetic heterogeneity that can lead to fatal ventricular arrhythmia and sudden cardiac death [1–3]. The clinical features are as follows: there is basic normal heart structure; the electrocardiogram ST segment of the right chest leads (V1–V3) is characteristically downward oblique or has a saddle-shaped elevation with or without right bundle branch block (RBBB); there is repeated syncope and sudden death when fatal ventricular arrhythmia (ventricular tachycardia or ventricular fibrillation) occurs; and most deaths occur in young men aged 30–40 years, who often have a family history of syncope or sudden death. In 1992, the Brugada brothers first summarized and reported similar clinical manifestations in eight patients with the disorder [4]. In Japan in 1996, Miyazaki et al. named it Brugada syndrome [5]. It is an inherited cardiac ion channel disease associated with an ion channel gene mutation [6] that can increase the risk of sudden cardiac death (SCD) [7, 8]. BrS is a rare and life-threatening arrhythmia disease in adults, with a relatively high prevalence rate of 0.12% [3, 9] in Southeast Asia, where the average age at the time of initial diagnosis or sudden death is 40 ± 22 years [10].

In recent years, there have been many studies worldwide on the pedigree inheritance and the pathogenic genes of BrS. Although 18 pathogenic genes are associated with BrS (SCN5A, SCN10A, SCN1B, SCN2B, SCN3B, GPD1L, RANGRF, SLMAP, CACNA1C, CACNA2D1, CACNB2, TRPM4, KCNE3, KCNH2, KCNJ8, KCNE5, KCND3, and HCN4), at present, it is considered a hereditary ion channel disease caused by a single gene [11]. Most of these variants are genes related to sodium, potassium, and calcium channels [12] and sodium voltage-gated channel alpha subunit 5 (*SCN5A*) (NM_198056.3), which encodes sodium channels and is the most common gene related to BrS [13]. Theoretically, the specificity and sensitivity of gene diagnosis can reach 100%. Therefore, prioritizing screening for the formation of SCN5A gene mutations and analyzing the association between families with BrS and gene mutations is crucial to improve the early diagnosis rate of BrS and implement early prevention for people carrying BrS genetic factors.

## 2. Materials and Methods

### 2.1. Research Subjects

This study was conducted on a Han family with BrS from Fujian, China; the family was comprised of 16 members in three generations, including eight males and eight females. The study was approved by the Ethics Committee of the Fujian Provincial Hospital. All recruited family members were informed, and they voluntarily signed an informed consent form before the clinical investigation. The BrS diagnostic criteria were as follows: in the second, third, or fourth intercostal chest leads (V1–V2) of the electrocardiogram, spontaneous type 1 Brugada was observed in more than one lead (J point elevation ≥2 mm with ST segment fornix-like elevation) [14]. Except for the use of sodium channel blockers, other conditions that may cause abnormal electrocardiogram (ECG) were excluded, such as the following: family history of polymorphic ventricular tachycardia, ventricular fibrillation, syncope or extreme dyspnea at night, SCD (<45 years old), downslope-type ECG changes in family members, and electrophysiological examination that can induce ventricular tachycardia/ventricular fibrillation [15].

## 3. Methods

### 3.1. Clinical Phenotype

The clinical phenotype data, medical history, ECG, 24-h ECG, cardiac color ultrasound, blood routine, blood coagulation, and biochemistry of the proband and related family members were collected.

### 3.2. DNA Extraction

Peripheral blood was collected from the proband (10 mL) and other investigated family members (2 mL) using ethylenediamine tetraacetic acid anticoagulation tubes, and genomic DNA was extracted according to the manufacturer's instructions (QIAamp DNA Blood Mini Kit, GER). The concentration and purity of the DNA were determined using a Nanodrop 1000 spectrophotometer (Nanodrop Technologies, USA).

### 3.3. Candidate Gene Location and Mutation-Screening Strategies

A genomic DNA library was built using TargetSeq® liquid phase probe hybridization capture technology independently developed by iGeneTech® (Beijing, China), and the promoter and exon regions (16.06 Mbp) of 508 genes related to genetic diseases were captured. PE150 was sequenced using IlluminaX10 or NovaSeq6000 platforms. Target genes related to hereditary heart disease included ANK2, CACNA1C, CACNA2D1, CACNB2, GPD1L, HCN4, KCND3, KCNE3, KCNE5, KCNH2, KCNJ8, PKP2, RANGRF, SCN10A, SCN1B, SCN2B, SCN3B, SCN5A, SLMAP, and TRPM4. Based on the BAM file results compared with genome reference sequences, single nucleotide variants and indels in the sequencing data were found and analyzed using software such as samtools, GATK, and ANNOVAR. Human genome information from the dbSNP, 1000G, Human Gene Mutation Database (HGMD), and ESP6500 databases was screened and annotated according to American College of Medical Genetics guidelines. Sequenced reads were referenced and compared with the hg19 sequence using the Burrow–Wheeler Aligner, and important data about these variants, such as gene information, mutation type, 1000G, and ESP6500 frequency, were obtained using polymorphism phenotyping (PolyPhen-2; https://genetics.bwh.harvard.edu/ppH2/), Sorting Intolerant from Tolerant (https://sift.jcvi.org/), MutationTaster (https://mutationtaster.org/), and other software for the prediction of mutation pathogenicity. Primer Premier 5.0 was used to design amplification primers for the upstream and downstream positions of the target mutation site, and the target region was amplified.

### 3.4. Sanger Sequencing Verification

The fragment of the suspected candidate mutation site was amplified using polymerase chain reaction (PCR) and verified by Sanger sequencing, and the corresponding sites for the proband and family members participating in the study were detected. The primers of the target sequence were designed using the Primer Premier 5.0 software, and the 288-bp target fragment was amplified using the GenBank SCN5A gene sequence (NM_000335.5) at the location of c.4313dup(p.Trp1439ValfsTer32) on exon 25. The primers used were F: 5ʹ-CCTCTTTCCCACAGAATGGA-3ʹ and R: 5ʹ- GGGAGCTGGTGCTCTACGTATCT-3ʹ. The annealing temperature was 59°C. Primers were synthesized by Synbio Technologies Co., Ltd. (Suzhou, China). The PCR product was amplified and purified using the Takara reagent and the target fragment PCR product was detected using an ABI3730XL sequencer.

### 3.5. SCN5A mRNA Expression Analysis Using Real-Time qPCR

RNA was extracted from five cases of p.Trp1439ValfsTer32 carriers and 10 cases of wild-type peripheral blood mononuclear cells (PBMC) according to the Tripure Isolation Reagent kit (#11667165001, Roche, Switzerland), and reverse transcription and real-time qPCR were performed to detect differences in *SCN5A* transcription levels. The RNA template was dissolved, added to primer mix, dNTP mix, DTT, reverse transcriptase (RT) buffer, HiFiScript, and RNase-free water, and kept on ice to prepare a 20 *μ*L reverse transcription reaction system. The reaction system was 4 *μ*L 2.5 mM dNTP mix, 2 *μ*L 2.5 mM primer mix, 7 *μ*L RNA template, 4 *μ*L 5 × RT buffer, 0.2 *μ*L 0.1 M DTT, 1 *μ*L 200 U/*μ*L HiFiScript, and RNase-free water to provide a final volume of 20 *μ*L. The solution was mixed by vortexing and briefly centrifuged to collect the solution from the tube wall to the bottom of the tube. It was incubated at 42°C for 50 min and then at 85°C for 5 min. After the reaction was complete, the mixture was centrifuged and placed on ice for cooling. The reverse transcription product can be directly used in PCR and real-time qPCR or stored at -20°C. The cDNA obtained by reverse transcription was diluted by 20 times and placed on ice to prepare a 20 *μ*L reaction system: 10 *μ*L 2 × UltraSYBR Mixture, 2 *μ*L 0.2 *μ*M Primers (F/R mix), and 8 *μ*L cDNA. A 40-cycle reaction program was performed with an added melting curve at 65°C–95°C (primers in Table 1).

## 4. Results

### 4.1. Clinical Phenotypes

**Figure 1:**
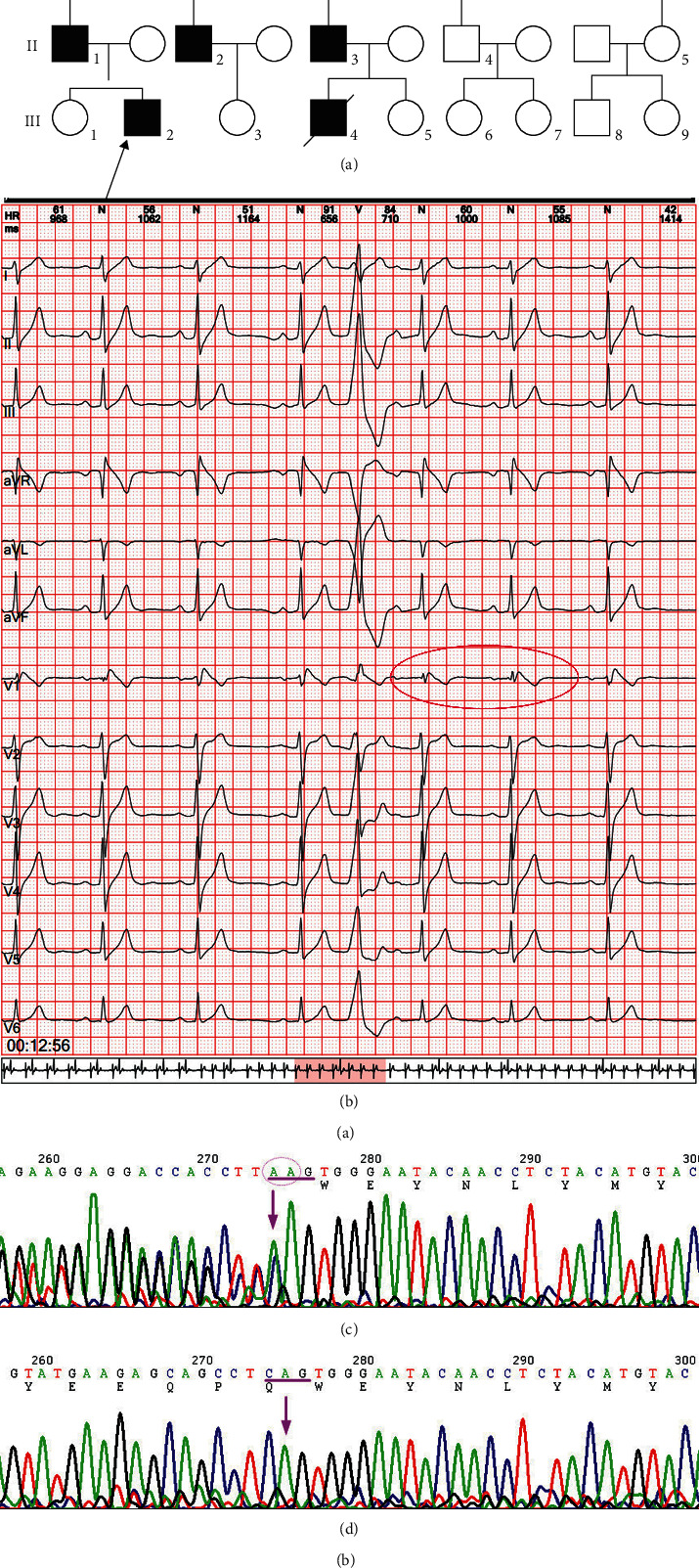
(a) Family map: black carries SCN5A (NM_000335.5) at c.4313dup (p.Trp1439ValfsTer32) mutation, the arrow is the proband, squares indicate males, and circles indicate females. (b) The proband (II1) 24 h ambulatory ECG: sinus rhythm; frequent ventricular extrasystole; intermittent first-degree atrioventricular block; intermittent abnormal ventricular repolarization (brugada wave in lead V1); continuous heart rate deceleration suggests moderate risk. (c) Sanger sequence diagram: c.4313dup (p.Trp1439ValfsTer32) mutant appeared in exon 25 of SCN5A gene. (d) Corresponding wild-type (WT).

**Figure 2:**
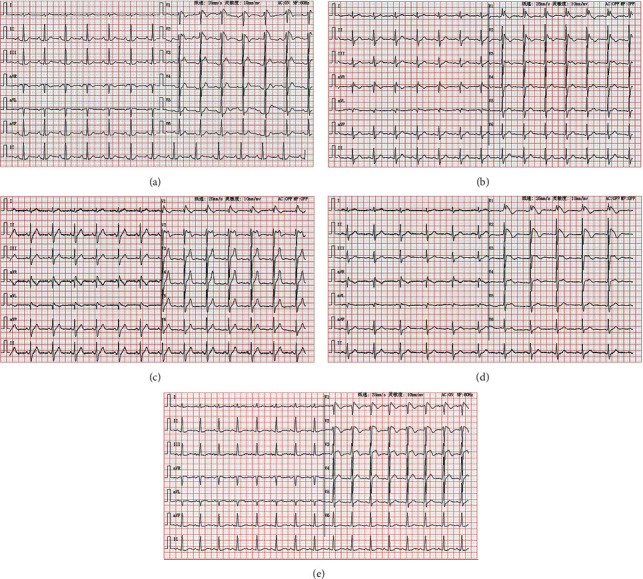
(a) Routine ECG manifestations of I1: sinus rhythm; complete right bundle branch block (RBBB); ST segment elevation (V1, V2), Brugada-like changes; (b) routine ECG manifestations of II2: sinus rhythm; brugada wave; (c) routine ECG manifestations of II3: sinus rhythm; the V1 and V2 leads showed Brugada wave-like changes; (d) routine ECG manifestations of III 2: sinus rhythm; the V1 and V2 leads showed Brugada wave-like changes; (e) routine ECG manifestations of III4: sinus bradycardia; incomplete RBBB? brugada syndrome? ST segment elevation (II, III, and aVF).

Six of the 16 family members were diagnosed with BrS (I1, II1, II2, II3, III2, III4, Figure 1(a): family diagram), all of whom were male. The proband (II1), 56 years of age, had a history of recurrent syncope for 3 years. Color Doppler echocardiography showed no obvious abnormalities in the cardiac structure and blood flow; however, a 24 h ambulatory electrocardiogram revealed sinus rhythm, frequent ventricular extrasystole, intermittent first-degree atrioventricular block, intermittent abnormal ventricular repolarization (V1 lead ST segment elevation >2 mm, “fornix-like” shape change, and *T* wave inversion, belonging to the type 1 Brugada wave (Figure 1(b)). Therefore, the proband was diagnosed with BrS. The nephew (III4) of the proband (II1) had a previous history of syncope, his ECG showed type 1 Brugada wave changes, and he died at the age of 18 years due to ineffective rescue from “sudden cardiac death.” The other four patients in the family (I1, II2, II3, and III2) showed no symptoms. Their ECG parameter 1 is shown in Figure 2 and Table 2. The other 10 family members had no previous history of cardiac morphological changes, syncope, ventricular fibrillation, or ventricular tachycardia.

### 4.2. Screening of Gene Mutations in BrS

Entire exons of the family members were sequenced and analyzed. Among single nucleotide polymorphisms and indels, the high-frequency mutations in the population and mutations in ExAC, gnomAD, and iGeneTechDB (local database, >10,000 samples) were removed. Subsequently, benign and likely benign mutations in the ClinVar and the synonymous variant mutation in Functional Human Genome Variation Society were removed. After screening, 440 variants remained. Twenty genes that had already been identified by ClinGen were searched (https://search.clinicalgenome.org/kb/affiliate/10045?page=1.size=All&search=), and only SCN5A was found. We found a mutation site in the proband (II1), which was a heterozygous mutation in exon 25 of *SCN5A* (NM_000335.5): c.4313dup(p.Trp1439ValfsTer32). There was a repeat base A at position 4313, resulting in a frameshift mutation leading to 32 incorrect codons followed by a stop codon. This locus has not been recorded in the ClinVar and HGMD databases, and it is a newly discovered mutation point (Figures 1(c) and 1(d)). Sanger verification revealed that I1, II1, II2, II3, and III2 with Brugada waves carried c.4313dup(p.Trp1439ValfsTer32) heterozygous mutations, whereas no c.4313dup(p.Trp1439ValfsTer32) variants were found in several other family members (I2, II4, II5, III1, III3, III5‒9) by Sanger verification.

### 4.3. Real-Time qPCR Results of SCN5A in Peripheral Blood

**Figure 3:**
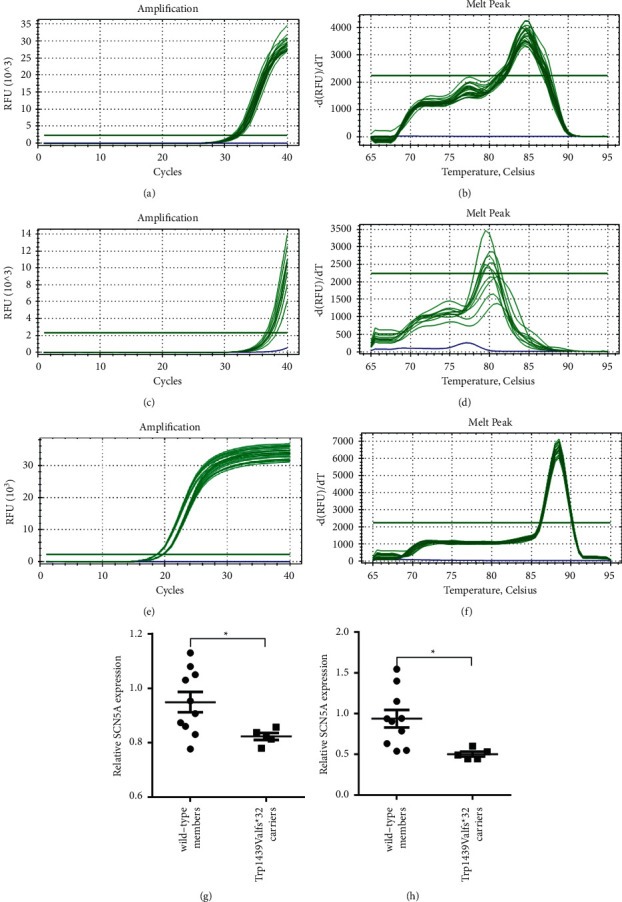
SCN5A mRNA expression analysis using real-time qPCR. (a‒f) The *SCN5A* amplification and dissolution curves. (g, h) The SCN5A expression differences of WT members (*n* = 5) and p.Trp1439ValfsTer32 carriers (*n* = 10).

RNA was extracted from the PBMC from five cases of p.Trp1439ValfsTer32 carriers and 10 cases of wild-type (WT), and the expression of SCN5A and the internal reference in the samples was detected using qPCR. The results showed that the expression of SCN5A mRNA in WT PBMCs was significantly higher than that in p.Trp1439ValfsTer32 carriers (Figure 3). This validates that the mutation can lead to nonsense-mediated mRNA decay theory or a truncated protein.

### 4.4. Prediction and Analysis of Bioinformatics

**Figure 4:**
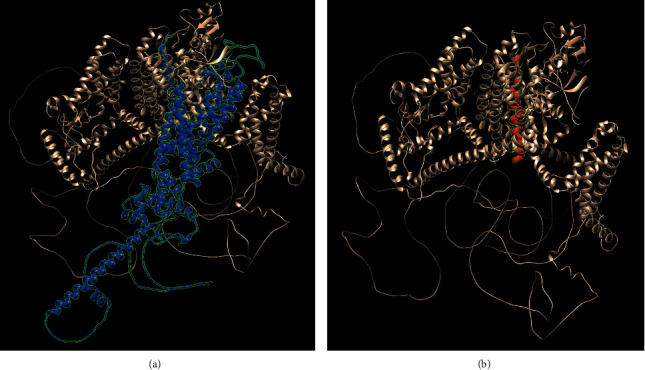
Tertiary structure of SCN5A WT and SCN5A c.4313dup (p.Trp1439ValfsTer32) mutant predicted using AlphaFold (https://alphafold.ebi.ac.uk/entry/Q14524) and shown in UCSF Chimera. (a) The blue region in SCN5A WT indicates the frameshift region resulting in 577 amino acids' deletion in p.Trp1439ValfsTer32 mutant. (b) The red region in SCN5A c.4313dup (p.Trp1439ValfsTer32) mutant (right) shows additional 32 amino acids caused by the frameshift.

The tertiary structure of the SCN5A WT and the SCN5A of c.4313dup (p.Trp1439ValfsTer32) mutant was predicted using AlphaFold (https://alphafold.ebi.ac.uk/entry/Q14524) and is shown in UCSF Chimera (Figure 4). The characteristics of the advanced structures were observed. The three-dimensional structure demonstrated that the frameshift region of the R1439fs mutant resulted in 577 amino acids' deletion in SCN5A WT. The addition of 32 amino acids to the SCN5A c.4313dup(p.Trp1439ValfsTer32) mutant caused a frameshift mutation.

## 5. Discussion

BrS is common in apparently healthy young men [16], with a male-to-female ratio of approximately 8 : 1, and the age of onset is mostly between 30 and 40 years of age [3]. Among BrS patients with cardiac arrest, males accounted for 64–94% [17]. Yamagata et al. surveyed 415 probands from Japan and found that almost all patients were male (403/415, 97%) [18]. The present survey also confirmed that men are more prone to the disease than women despite the similar genetic transmission probability of mutations between men and women, as BrS patients in the family were all men. A potential mechanism associated with sex morbidity can be that a more significant action potential gap exists in men than in women, mediated by the transient outward potassium current (*I*_to_), in the epicardium of the right ventricle [19]. In the wedge-shaped model of arterial perfusion in male tissue, further activation of *I*_to_ caused the repolarization of right ventricular epicardial action potential to more negative potentials at the end of the first stage, which promoted the loss of the action potential dome and development of action potential reentry in the second stage [20], implying a higher risk of type 1 Brugada wave and ventricular fibrillation in men than in women [21]. Nevertheless, no suitable sex-specific risk predictors have been found in the current study [22], which may be due to the various inducing factors and circumstances of SCD in BrS patients [23].

In this BrS family, the proband (II1) and his nephew (III4) repeatedly fainted, and III4 died suddenly after exercise, whereas the other four BrS patients (I1, II2, II3, and III2) did not have any symptoms. According to several registered studies of the FINGER trial, about 67% of BrS patients are asymptomatic; the disorder is usually found accidentally during examination and BrS can be asymptomatic for life [21]. A few people showed dyspnea, syncope, and palpitations at night. The annual incidence of arrhythmic events is 0.5% [21] and is often characterized by ventricular tachycardia, ventricular fibrillation, or SCD. Approximately 20% of patients with BrS also have supraventricular arrhythmias, including atrial flutter, fibrillation, and preexcitation syndrome (such as Wolff-Parkinson–White syndrome) [24, 25]. Arrhythmia caused by BrS may be multifactorial, and abnormal pathophysiological mechanisms in its genetic background may be important. For example, a study suggested that fibrosis on the epicardial surface and decreased expression of the right ventricular outflow tract gap junction are the mechanisms of arrhythmia caused by BrS [26]. BrS occurs in 0.015% of the world's population, with a lower incidence in Western countries and a higher incidence in Southeast Asia. In Thailand, the proportion of patients who die of BrS in the natural population is as high as 4–10/10,000, which is considered the main cause of sudden death among young people and is referred to as inexplicable sudden death syndrome [27, 28]. An epidemiological study showed that approximately 50% of all patients with nonstructural heart disease have SCD caused by BrS, which is the most common cause of SCD among young people in South Asia [29, 30]. Malignant syncope in patients with spontaneous type 1 Brugada waves can lead to a high risk of SCD. In contrast, asymptomatic patients with spontaneous type 1 Brugada waves usually follow a benign clinical process [31]. The history of syncope is closely related to the increased risk of SCD, which is about four times higher than that of asymptomatic patients [32, 33]. Family history has also been suggested as a major risk factor for cardiac accidents in patients with BrS. The annual incidence of cardiac accidents in first-degree relatives with early SCD family history is 3.1%, while that in BrS patients without SCD family history is 1.3% [34]. However, some studies have denied its usefulness in predicting major arrhythmia events [35]. In addition, *SCN5A* mutation is an important predictor of cardiac events, and the risk ratio of SCN5A (+) to SCN5A (−) was 2.0 (*P*=0.045) [18]. It is obvious that the proband belongs to the high-risk group for cardiac incidents and needs clinical intervention to avoid the occurrence of cardiac incidents.

Approximately 14–26% of BrS patients have mutations in SCN5A, which is considered the most common BrS-related gene [18, 36], and the prevalence of SCN5A mutation in BrS patients in the Chinese Han population is 7.5% [37]. To date (March 1, 2022), the HGMD (https://www.hgmd.cf.ac.uk/ac/gene.php?gene=SCN5A) contains 852 clinically significant mutations, including missense or nonsense (689), splicing (38), small deletions (79), small insertions (35), small indels (7), gross deletions (3), and complex rearrangements (1). Among them, 381 SCN5A mutation sites were identified as causing BrS and 33 were suspected to lead to the disease. The SCN5A gene is located on chromosome 3p21, encodes a protein consisting of 2016 amino acids, and is a member of the voltage-gated sodium channel family [38]. In BrS patients with SCN5A mutation, a loss of function encoding cardiac sodium channel *α*-subunit NaV1.5 is observed, followed by a decrease in sodium current [39]. As a result, the *I*_to_ potential difference between the endocardium and adventitia of the ventricle increased, and the ECG showed an elevated *J* point and ST segment. After our newly discovered position c.4313, 25 frameshift mutations were identified by querying the ClinVar database (https://www.ncbi.nlm.nih.gov/clinvar), of which 10 frameshift mutations were identified as pathogenic, 9 were considered likely pathogenic, and two were considered likely pathogenic/pathogenic. Nine of the twelve pathogenic mutations are reportedly associated with BrS. According to the American College of Medical Genetics and Genomics rating, we found that the c.4313dup (p.Trp1439ValfsTer32) was pathogenic.

Functional studies of two missense mutations and one insertion mutation near c.4313dup (p.Trp1439ValfsTer32) were performed. Six et al. identified the c. C4313T (p.P1438L) mutation in SCN5A in patients with BrS. In the overexpressed WT (Nav1.5WT) and mutant (Nav1.5P1438L) tsA201 cell lines of the *SCN5A* gene, the normal sodium current of WT (Nav1.5/WT) cells was rapidly activated and inactivated, whereas the opposite was observed for the mutant channel (Nav1.5/P1438L), which did not cause any current. The transfected cells had no inward current with the P1438 mutant [40]. The cardiac Nav1.5 encoded by SCN5A consists of four homologous domains (DI‒DIV), which are connected by intracellular connectors, and each domain contains six transmembrane fragments (S1–S6). Mutation of c. A4342C(p.I1448L) in exon 25 of SCN5A can also lead to BrS, which can cause loss of DIII-S5/S6 function. One study found that nearly 10% of clinically suspected BrS patients had mutations in the channel pore/selectivity filter, that is, the S5 and S6 segments and interconnected *P*-loops. In this study, it was uncertain whether BrS patients with porous mutations experienced more signs and symptoms than those with mutations located in other domains [41]. Moreover, the study found that c.C4313T (p.P1438L) of exon 25 is located at DIII-S5/S6, which is near our mutation point. We speculate that the c.4313dup (p.Trp1439ValfsTer32) mutation results in loss of function of DIII-S5/S6, which is localized in the pore region and the selectivity filter of the channel. The P-loop between segments S5 and S6 constitutes the central pore-forming region that determines the ion selectivity of the channel. Therefore, we believe that c. 4313dup (p.Trp1439ValfsTer32) can induce loss of function of Nav1.5. In an infant with BrS with rapid ventricular tachycardia induced by febrile convulsion, a novel heterozygous 1-bp insertion mutation (InsG) in the nt4392-4396 position of the *SCN5A* coding sequence was found, which led to the destruction of the reading frame of SCN5A, resulting in a premature stop codon (1483X) and a truncated polypeptide [42]. However, 4196delA (V1397X) and 5280delG (1768X) mutants did not exhibit any sodium current in cell electrophysiological experiments [36, 43]. This experiment suggests that mutation 1483x might not produce sodium current. Bioinformatics prediction demonstrated that the c.4313dup(p.Trp1439ValfsTer32) mutation affected the expression of SCN5A. The frameshift region of the c.4313dup(p.Trp1439ValfsTer32) mutant led to the deletion of 577 amino acids in WT *SCN5A*, and the addition of 32 amino acids to the c.4313dup(p.Trp1439ValfsTer32) mutant led to a frameshift mutation, resulting in truncated protein products. Alternatively, the mutant allele may become invalid because the mutant mRNA is degraded by nonsense-mediated decay [44].

To date, the pathogenesis of BrS is not well understood and effective drugs to cure BrS are lacking. Implantable cardioverter defibrillator (ICD) is considered the first-line therapy to prevent SCD in high-risk BrS patients [45]. In a 20-year follow-up of patients with spontaneous or drug-induced type 1 Brugada electrocardiographic manifestations, ICD implantation treated the potentially fatal arrhythmias of 17% of patients, suggesting that ICD is an effective strategy for the treatment of BrS [46]. At present, there is a consensus on the management of asymptomatic BrS patients. Electrophysiological study (EPS) should be conducted for asymptomatic patients with a family history of Brugada-related SCD, but there is no clear recommendation for asymptomatic patients with a negative family history [47]. ICD therapy should be recommended for asymptomatic BrS patients who meet the following three signs: spontaneous or drug-induced type I Brugada ECG pattern, SCD family history secondary to BrS, and the inducibility of ventricular arrhythmias that can be proven in EPS [48].

## 6. Conclusion

Detection of the SCN5A gene is helpful for the diagnosis of BrS, and the newly discovered SCN5A c.4313dup (p.Trp1439ValfsTer32) mutation enriches the mutation spectrum of the SCN5A gene. Evaluating the risk factors of patients with BrS who have been diagnosed is important and conducive to the individualized treatment of BrS and the prevention of adverse cardiac events.

## Figures and Tables

**Table 1:** Design of reverse transcription primers for qPCR detection of *SCN5A* in peripheral blood.

Primer name	Primer sequence
SCN5A mutation site pre-qPCR F^*∗*^	5′-CCAGACAGAGGGAGACTTGC-3′
SCN5A mutation site pre-qPCR R^*∗*^	3′-CTGGAGTCCACAGCTGCATA-5′
qPCR after SCN5A mutation site F	5′-CGCCTACGTGATGAGTGAGA-3′
qPCR after SCN5A mutation site R	3′-GTCGGCGAGATCTTCACTGT-5′
hGAPDH F	5′-CAAGGTCATCCATGACAACTTTG-3′
hGAPDH R	3′-GTCCACCACCCTGTTGCTGTAG-5′

^*∗*^F as forward and R as reverse.

**Table 2:** ECG characteristics of five patients with Brugada syndrome.

Features	R-R interval (s)	P wave (mV, s)	P-R interval (s)	QRS complex (mV, s)	ST segment (s)	T wave (mV)	U wave	QT interval (s)	R_V5_/S_V1_ (mV)	R_V5_ + S_V1_ (mV)
Normal amplitude	—	0.25	—	1.60 for R peak	—	0.1–0.8	May not be observed because of its small size	—	＜2.50/1.00	＜3.50
Normal duration	0.6–1.2	0.08–0.11	0.12–0.20	0.06–0.10	0.05–0.155	0.05–0.25	Unknown	0.35–0.44	—	—
I1	0.72	0.102	0.198	0.120	0.082 s	0.139 s	No	0.338	2.12/0.93	3.05
II2	0.72	0.094	0.174	0.104	0.082 s	0.149 s	No	0.354	1.28/0.05	1.33
II3	0.85	0.098	0.152	0.116	0.036 s	0.106 s	No	0.334	1.37/0.00	1.37
III2	0.88	0.114	0.186	0.138	0.152 s	0.157 s	No	0.396	1.25/0.00	1.25
III4	1.1	0.098	0.154	0.100	0.154 s	0.164 s	No	0.394	1.49/0.76	2.25

## Data Availability

The de-identified participant data will not be shared.
